# A Convex Formulation for Magnetic Particle Imaging X-Space Reconstruction

**DOI:** 10.1371/journal.pone.0140137

**Published:** 2015-10-23

**Authors:** Justin J. Konkle, Patrick W. Goodwill, Daniel W. Hensley, Ryan D. Orendorff, Michael Lustig, Steven M. Conolly

**Affiliations:** 1 Department of Bioengineering, University of California, Berkeley, CA, United States of America; 2 Department of Electrical Engineering and Computer Sciences, University of California, Berkeley, CA, United States of America; University of Pécs Medical School, HUNGARY

## Abstract

Magnetic Particle Imaging (mpi) is an emerging imaging modality with exceptional promise for clinical applications in rapid angiography, cell therapy tracking, cancer imaging, and inflammation imaging. Recent publications have demonstrated quantitative mpi across rat sized fields of view with x-space reconstruction methods. Critical to any medical imaging technology is the reliability and accuracy of image reconstruction. Because the average value of the mpi signal is lost during direct-feedthrough signal filtering, mpi reconstruction algorithms must recover this zero-frequency value. Prior x-space mpi recovery techniques were limited to 1d approaches which could introduce artifacts when reconstructing a 3d image. In this paper, we formulate x-space reconstruction as a 3d convex optimization problem and apply robust *a priori* knowledge of image smoothness and non-negativity to reduce non-physical banding and haze artifacts. We conclude with a discussion of the powerful extensibility of the presented formulation for future applications.

## Introduction

Magnetic Particle Imaging is a novel, safe, sensitive, high-contrast, and fast imaging modality [1–6] with many potential applications in medical imaging including angiography, cell therapy tracking, cancer imaging, inflammation imaging, and temperature mapping [5, 7, 8]. The mpi technique detects only magnetic particles and derives no signal from tissue, which gives mpi unique contrast that is best compared with tracer imaging modalities such as nuclear imaging. This is in contrast to Computed Tomography (ct) and Magnetic Resonance Imaging (mri), which are primarily anatomical imaging techniques. The physics and hardware required for mpi are completely distinct from existing medical imaging modalities, and mpi images cannot be acquired using mri systems.

mpi produces images of magnetic nanoparticle (mnp) concentrations by detecting the nonlinear magnetic response of an mnp distribution to time varying magnetic fields. A strong static magnetic field gradient or selection field saturates all mnps in the field of view (fov) except for a region near the center of the fov called a field-free region (ffr), which can be either a field-free point (ffp) or field-free line (ffl). A second low-frequency, time-varying (*e.g*., sinusoidal) homogeneous magnetic field called the drive field excites the mnps. The drive field translates the ffr, which causes a flip in magnetization when the ffr passes over the mnps. This flip in magnetization induces a signal in a receive coil. The fov is extended using a slowly varying focus field or shift field.

To reconstruct the received signal into an image, two distinct approaches to image reconstruction have been demonstrated: system function reconstruction [1, 2, 9–15] and x-space reconstruction [3–5, 16–19]. The system matrix method measures or simulates the mnp response in a specific mpi system with a pre-defined trajectory to form a system matrix. The system matrix is then used to reconstruct an image. In contrast, x-space methods use an image space continuity algorithm which do not require any simulation or pre-characterization measurements of the mnp response. However, current x-space continuity algorithms operate sequentially on a single 1d line at a time and do not take advantage of information along the two perpendicular axes.

Optimization approaches have been used for image reconstruction in mri and ct to increase imaging speed, reduce image artifacts, and reduce dose [20–28]. For example, some techniques formulate the mri and ct reconstruction process using reliable *a priori* knowledge regarding the governing physics and imaging process such as smoothness, non-negativity, data consistency, sparsity, and multiple imaging channels [20, 21, 24, 25].

These optimization approaches can be applied to mpi, where reliable *a priori* information exists and can be used to improve reconstruction accuracy. In this paper we formulate the mpi 1d, 2d, and 3d x-space dc (direct current or zero-frequency) recovery and image stitching processes as a convex optimization for the first time while enforcing knowledge that the image must be both smooth and non-negative. This new optimization approach utilizes additional information along the two axes perpendicular to the excitation axis to improve on our previous x-space reconstruction.

## Theory

The x-space systems theory for mpi is described in [3–5, 16–18]. The mpi signal equation and point spread function (psf) were derived using the assumption that mnps instantaneously align with an applied magnetic field [16, 17]. The systems theory was then extended to include the first-harmonic direct-feedthrough filtering necessary in real mpi systems [18]. The filtered information was found to correspond to a loss of spatial dc information. X-space theory has been used to prove analytically and experimentally that this dc loss can be reversed to restore linearity and shift invariance in mpi [18].

In this work, we demonstrate that the mpi x-space reconstruction process can be improved in 2d and 3d using convex optimization with the following *a priori* information: the mnp distribution is non-negative and the mnp distribution is smooth. The validity of these assumptions in mpi systems is described below.

### New a priori information: 2d and 3d smoothness and non-negativity

mpi images the density of mnps convolved with a strictly positive psf. Thus it is not possible for the mpi image, the convolution of two positive functions, to contain negative values except for those produced by noise. Enforcing non-negativity during image reconstruction is then a physically justifiable assumption.

The reconstructed mpi image must also be smooth due to a smooth mpi psf. The native mpi image is a convolution of the physical mnp distribution with the smooth psf and is thus smooth. If the sampling of the native image adheres to the Nyquist limit (determined by the band-limited psf), the reconstructed image must also be smooth.

In a multi-dimensional image reconstruction algorithm, one efficient method of incorporating non-negativity and smoothness is through convex optimization methods, which can solve for convex objectives (e.g., the sum of a data consistency term and a 3d smoothness term) and convex constraints such as non-negativity. The use of these additional terms and constraints enforces a globally optimal solution that adheres to the physics of the mpi process, thereby increasing image conspicuity.

## Materials and Methods

**Fig 1:**
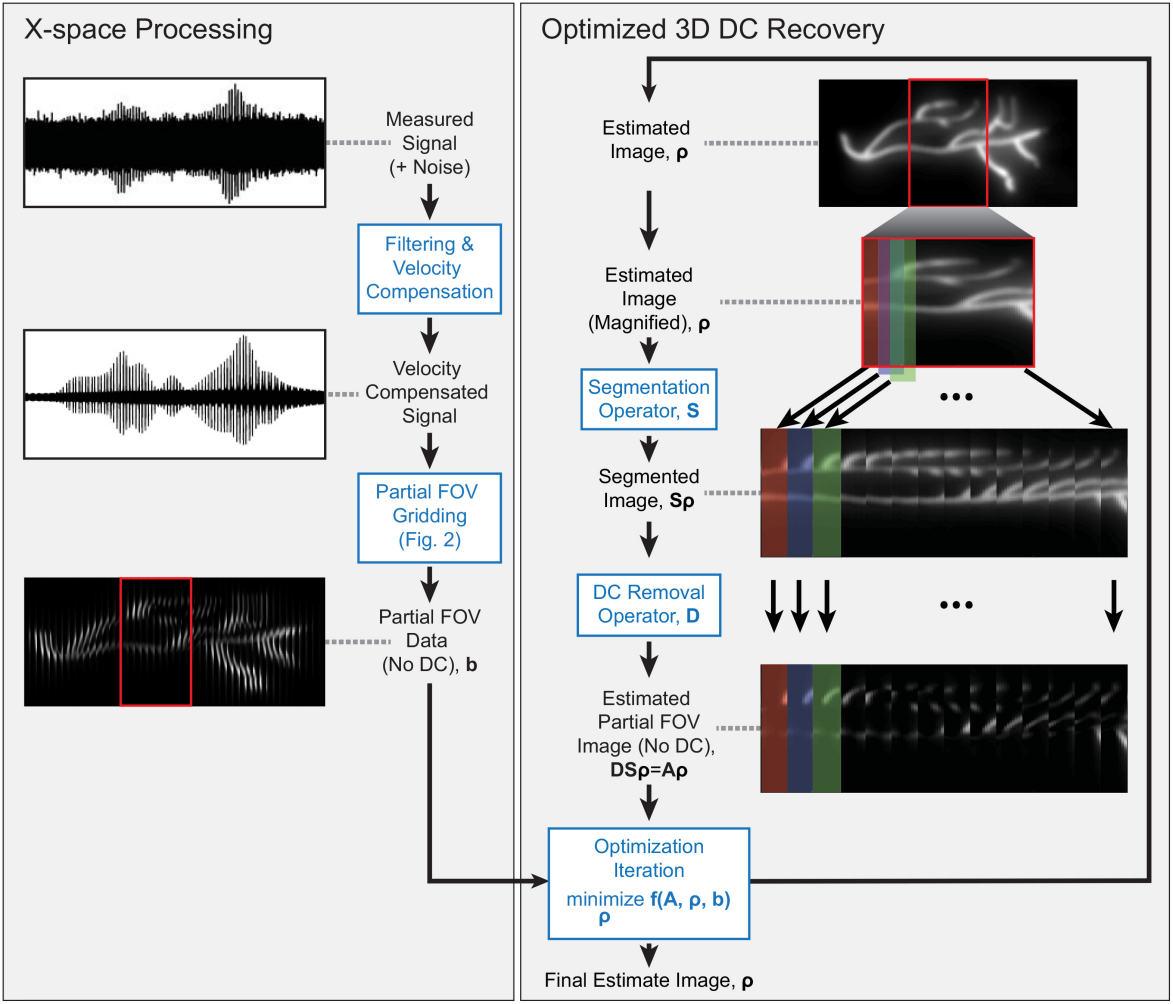
Experimental data illustrating proposed image reconstruction. **(Left)** The measured signal is filtered and velocity compensated before gridding to partial fov images. The partial fov) images become the input to the optimization problem. **(Right)** The optimization problem formulation of dc recovery is illustrated. The forward model **A** consists of the **S** and **D** operators, where **S** is the segmentation operator and **D** is the dc removal operator. The initial estimated image is the zero vector, ***ρ***_0_ = **0**. The estimated image, ***ρ***, is calculated and updated with each step of the iterative proximal gradient solver [29]. The optimization problem is formulated in Eq 6.

The reconstruction pipeline can be broken down into two serial processing steps: x-space processing and optimized dc recovery (see Fig 1). The x-space processing filters and velocity compensates the raw data acquired by the analog to digital converters (adcs) and interpolates the data into partial fovs. The optimized dc recovery then minimizes the residual error between partial fov data and estimated partial fovs. The estimated partial fovs are calculated via a forward operator on an estimated image. The linear operators that constitute the forward model are represented by sparse matrices and/or functions specific to a particular mpi pulse sequence. The optimization problem includes *a priori* information such as smoothness and non-negativity. The problem is solved with a standard gradient descent-based algorithm using a matrix-free formulation which is fast, robust to noise, and memory efficient. We describe these steps in detail below.

### X-space processing

X-space processing prepares the raw signal for the optimization problem and reduces the size of the dataset via three main steps: filtering, velocity compensation, and partial fov gridding. These steps remain identical to the previously reported x-space reconstruction and are illustrated in the left column of Fig 1 [16, 18].

The filtering step of x-space processing recovers signal phase and reduces noise. Phase correction filters reverse the phase distorted by the hardware filter chain. High pass filters remove any remaining direct-feedthrough at the fundamental frequency. Digital harmonic filtering removes signal outside a specified bandwidth of the received harmonics in the Fourier domain.

After filtering, velocity compensation is performed by normalizing the signal intensity to the instantaneous ffr velocity as required for x-space reconstruction [16, 17].

**Fig 2:**
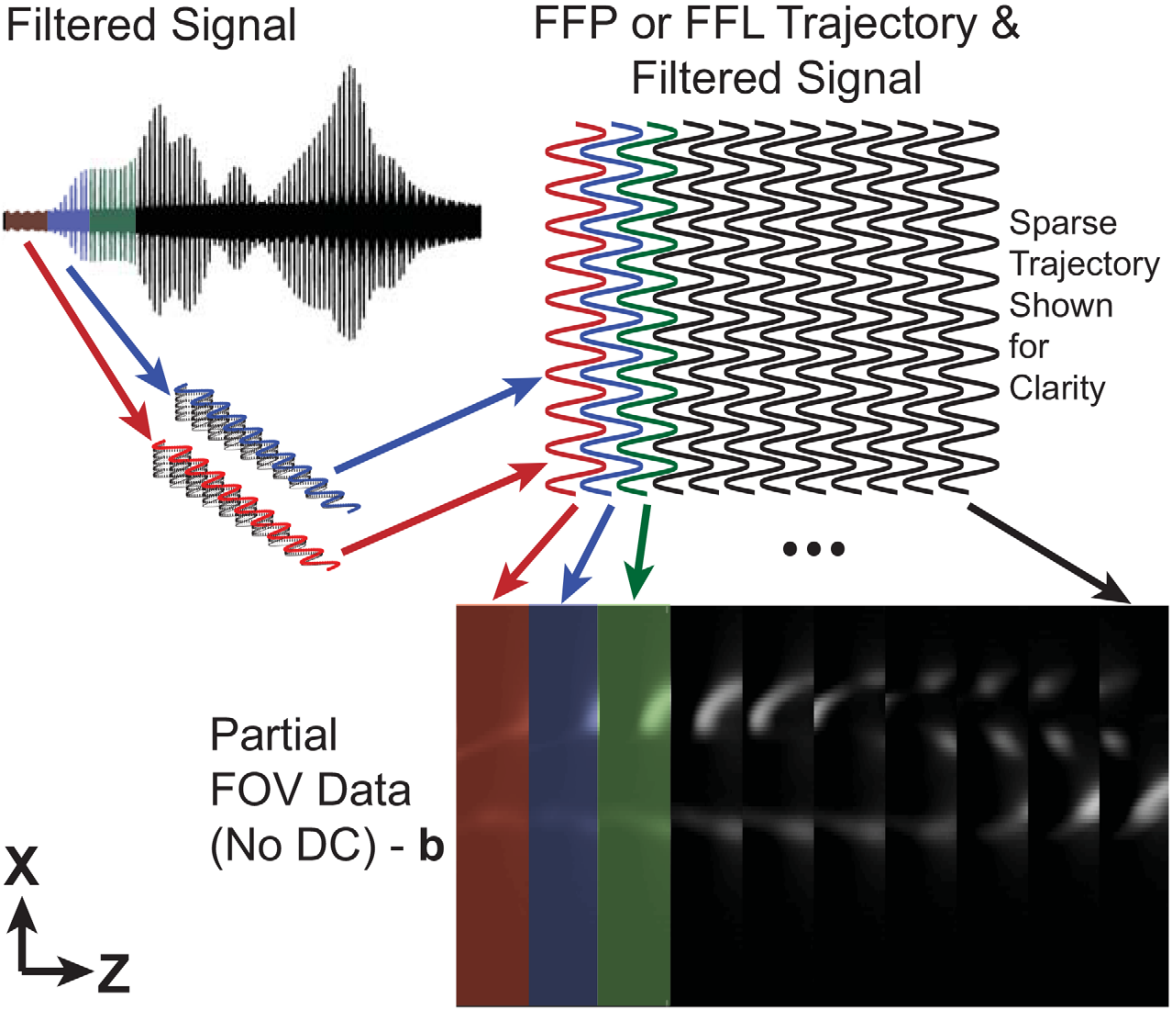
Partial field of view gridding detail. The received signal is interpolated to partial fov images using the ffr trajectory. Each *x*-axis traversal is broken into a separate partial fov image. Varying colors delimit each partial fov image. The sinusoidal pattern in the trajectory is formed due to the simultaneous *x*-axis shift field and the *z*-axis drive field.

The signal is then gridded into partial fov images as detailed in Fig 2. Image data is interpolated onto a discrete grid using the known trajectory of the ffr. The trajectory is redundant and creates overlapping partial fov sub-images where one partial fov is defined as the spatial extent the ffr travels due to the drive field. The resulting partial fov data is missing some unknown portion of the dc component in the partial fov image (along the *z*-axis in Fig 2) due to direct feed-through filtering in hardware [10, 18]. In this work, the remaining unknown dc component is removed by filtering dc to zero.

Averaging during interpolation improves the final image signal to noise ratio (snr) and also reduces the storage size of the processed partial fov data when compared to the raw data acquired by the adc. The original vector of raw data for the coronary phantom images shown in this work contain 740 million values of data (6GB) while the partial fov data, **b**, contains 14 million values (112MB). Gridding reduced the memory size and optimization problem input size by a factor of 50 in this example and simplified the forward model employed in the optimization problem. Problem size reduction depends on spatial density of the sampled trajectory and the partial fov interpolation density.

### Linear Forward Model

A linear forward model describes the splitting of an image into partial fovs and the dc signal loss due to filtering (see Fig 1, right side). The forward model is a simplified description of the imaging process. The linear forward model allows specification of the data consistency term of the optimization problem formulated in Eq 6.

The forward model includes two operators, segmentation **S** and dc removal **D**. **S** is the segmentation operator, which breaks the image into overlapping partial fov images:

s=[IsIrIsIsIrIs⋱] (1)

where **I_s_** is an identity matrix the size of the overlap, *s*, between adjacent partial fov images. **I_r_** is an identity matrix the size of *r* = *p* − 2*s* where *p* is the width of partial fov. This definition is specific to the problem with the image vectorized along the rows and partial fovs shifted by an integer number of pixels.

The operator, **D**, removes the average along the drive field direction (here the *z*-axis) of the partial fov:

$$\text{D}=[\begin{array}{cccc} \text{R}\\ & \text{R}\\ & & \ddots \\ & & & \text{R} \end{array}]$$ (2)

where

$$R=I_{p}-\frac{1}{p}.$$ (3)

This operation is equivalent to subtracting the dc component in the spatial Fourier domain.

Operators **S** and **D** are composed to form the forward model of the mpi system, **A**:

A=DS (4)

where $A\in R^{m\times n}$ is a matrix, *n* is the product of the dimensions of the resulting image, and *m* is the product of the dimensions of the input partial fov images. Both operations **S** and **D** are sparse, and their composition results in an **A** matrix that is sparse and has a block diagonal-like structure. The forward model is then described by:

b=Aρ (5)

where $b\in R^{m}$ is the input data of vectorized partial fovs from the scanning system and $\rho \in R^{n}$ is the vectorized image of mnp density convolved with the system psf. The vectors are built by stacking the rows of the image or the rows of the partial fov. Note that no assumptions regarding nanoparticle behavior were made except that the nanoparticles respond to the instantaneous position of the ffr.

### Reconstruction Formulated as a Convex Optimization

Because we have represented the imaging process as a set of linear operations, we are able to estimate the native mpi image by solving a convex optimization, expressed below. A convex optimization formulation guarantees that any minimum reached is a global minimum [30].

$$\begin{array}{l} \underset{\rho }{\text{minimize}}\;\;{∥A\rho -b∥}_{2}^{2}+\alpha {∥\rho ∥}_{2}^{2}+\beta_{i}{∥\nabla_{e_{i}}\rho ∥}_{2}^{2}\\ \text{subject}\;\text{to}\;\;\rho ≽0 \end{array}$$ (6)

where ≽ denotes element-wise inequality for non-negativity, ***ρ*** and **b** are as described in Eq (5), *α* is a Tikhonov regularization parameter, *β*_*i*_ are smoothness parameters, and **e**_*i*_, *i* ∈ {1, 2, 3} is one of the three coordinate axis basis vectors. The image non-negativity constraint improves the general robustness of the dc recovery. As noted above, the addition of smoothness and non-negativity terms are justified by *a priori* knowledge of the physics.

The smoothness terms *β*_*i*_ (which penalize the spatial image gradients) and the Tikhonov regularization *α* increase the stability of the image reconstruction. Tikhonov regularization is used to better condition a problem. This is true of our problem as the Tikhonov term regularizes the singular value associated with dc, originally in the nullspace, by forcing the optimization to choose an image estimate with the lowest total dc value. For our problem, this has a strong connection with *a priori* knowledge that real mpi images are tortuous and sparse.

Eq 3 can be restated more generally:

$$\begin{array}{l} \underset{\rho }{\text{minimize}}\;\;{∥T\rho -w∥}_{2}^{2}\\ \text{subject}\;\text{to}\;\;\rho ≽0 \end{array}$$ (7)

where

$$T=[\begin{array}{c} A\\ \sqrt{\alpha }\;I\\ \sqrt{\beta_{i}}\;\nabla_{e_{i}} \end{array}]\;w=[\begin{array}{c} b\\ 0\\ 0 \end{array}]$$ (8)

In this form, the image reconstruction problem is a basic least squares problem subject to a non-negativity constraint. Many tools for solving this basic form of non-negative least squares are available in common scientific computing platforms; however, these tools do not support using matrix-free operators to solve optimization problems. Our motivation to use matrix-free methods is described in the next section. We implemented a proximal gradient algorithm (Fast Iterative Shrinkage-Thresholding Algorithm (fista)) using matrix-free operators, where the proximal operator is a projection onto the non-negative orthant [29, 31]. With this solver, we can compare the practical computational advantages and disadvantages of using matrix-free operator formulations over matrix formulations.

### Linear Operator Representation

The image reconstruction problem can be complicated by the need to store very large matrices. Simply storing these matrices can be a challenge, even with considerable sparsity of approximately 1:10^5^. For example, the matrix **A** in Eq 3 requires approximately 32GB of memory for the 3d data sets acquired in this work when stored in a standard sparse form.

Instead of storing sparse matrices, matrix-free operators can be used. With matrix-free operators, the matrix-vector multiplication is encoded as a function, and no actual matrix is stored. These matrix-free operator methods are used in mri, ct, and geology to reduce the storage requirements of imaging problems [26, 32, 33].

In practice, there are two challenges in converting a given matrix formulation into the equivalent matrix-free operator formulation. First, one must derive a function for the forward linear map (**A** ***ρ***). Then, to solve an optimization problem using this forward model, one must derive a function for the corresponding adjoint (**A** ^⊤^ **b**). Here, matrix-free operator formulations for both the dc removal operator, **D**, and the splitting operator, **S**, and by composition, **A**, were developed. The functional forms can be checked for correctness by operating on the identity (returning the linear map in its finite, dense matrix form) and through the dot-product test [33]. As noted in the results section, going to matrix-free operator methods has improved reconstruction time seven-fold and greatly reduced ram requirements.

### Imaging Phantoms

**Fig 3:**
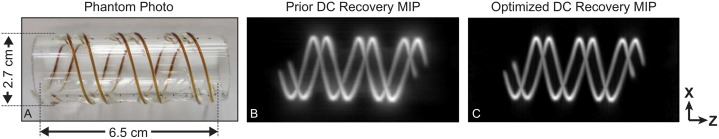
Experimental MPI data from a double helix phantom. The 3d dataset was reconstructed using the previous dc recovery method and the proposed method. Both datasets are shown as maximum intensity projection images with no deconvolution. Images reconstructed with the proposed method contain less background haze and fewer artifacts. The imaging phantom was constructed by wrapping two 0.6mm id tubes injected with Micromod Nanomag mip mnps around an acrylic cylinder of od 2.7 cm. The total imaging time was 10 min with a fov of 4.5 cm by 3.5 cm by 7.5 cm (*x*,*y*,*z*).

To demonstrate the reconstruction method using our mpi system, two imaging phantoms were created. A double-helix phantom shown in Fig 3 was fabricated from two 0.6mm inner diameter tubing segments injected with mnps (Micromod Nanomag-MIP 78-00-102, Rostock, Germany). These tubing segments were wound around a 2.7 cm acrylic cylinder with a total length of 6.5 cm.

**Fig 4:**
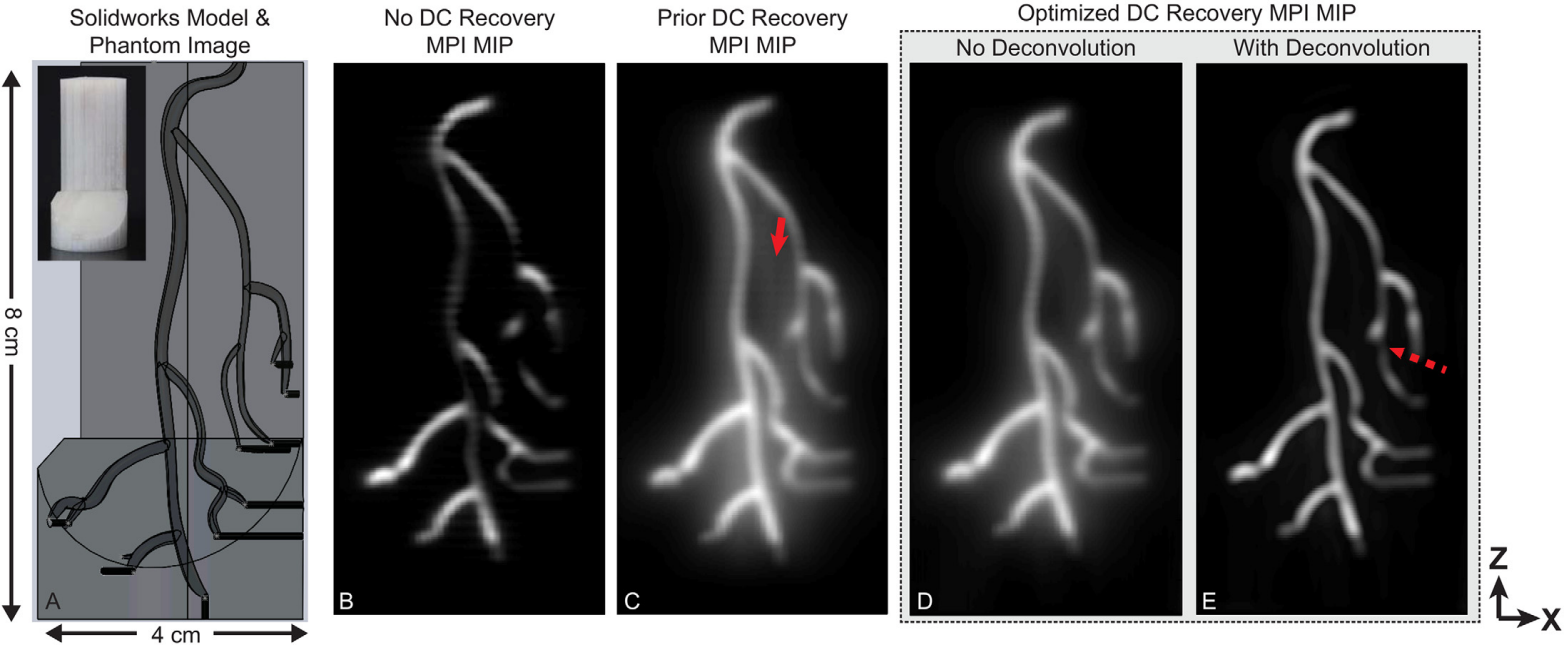
Experimental MPI data from a coronary artery phantom. Images were reconstructed with the proposed reconstruction formulation and contrasted to the previous 1d dc recovery as well as no dc recovery. The imaging phantom was created by 3d printing an abs plastic coronary artery model. The reconstructed 3d dataset is shown as maximum intensity projection images. With no dc recovery, many image intensity dropouts are evident. These dropouts are corrected with dc recovery algorithms. The optimized 3d recovery contains fewer artifacts (solid arrow) and less background haze than the prior algorithm. Light deconvolution can be used to remove remaining background haze present in the reconstructed signal; however, deconvolution can lead to image dropouts (dashed arrow). The total imaging time was 10 min with a fov of 4.5 cm by 3.5 cm by 9.5 cm (*x*,*y*,*z*).

A coronary artery phantom 3d model with approximately human sized features was designed in SolidWorks (Dassault Systems, Maltham, MA). The arteries formed cavities in a cylindrical part. The part was printed on a 3d printer (Afinia H480, Chanhassen, MN). The 3d model is shown in Fig 4. The phantom was designed with 1.8mm by 2.3mm maximum diameter arteries that were approximately ellipsoidal. Injection holes (shown in black) had a diameter of 1.0mm and were filled with Micromod Nanomag mip mnps diluted 4:1 with deionized water.

**Fig 5:**
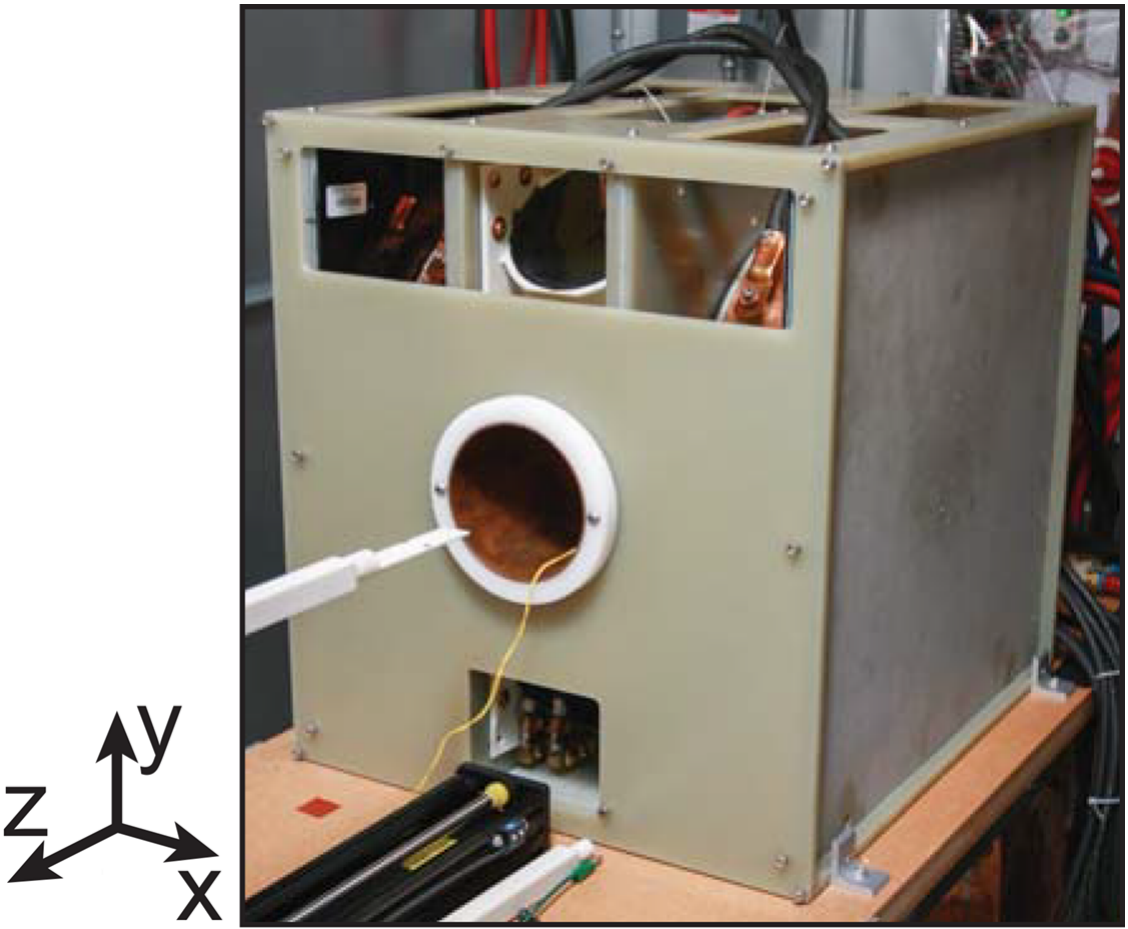
Field free point MPI system photo. This 7Tm^-1^ ffp mpi system was used to experimentally demonstrate the effectiveness of the 3d optimized reconstruction.

The phantoms were imaged with the ffp imaging system shown in Fig 5. The images were reconstructed using the formulation in Fig 1. The optimization problem formulated in Eq 4 was solved via a proximal gradient method developed in Matlab [29]. To reconstruct the image, 15 harmonics were used, for a total bandwidth of 300 kHz.

We included comparisons between native x-space reconstructed images and mildly deconvolved images in the results. Deconvolved images were generated using 3d Wiener deconvolution [34]. The estimated psf returned by blind deconvolution, seeded with a calculated theoretical mpi psf, was used in the Wiener deconvolution. Deconvolution was applied after x-space reconstruction and independent of the optimization.

## Results

In Fig 3, the proposed reconstruction is compared to the previous x-space algorithm using experimental mpi data from a double helix phantom. Fewer banding artifacts and haze are present with the proposed algorithm. No deconvolution is used. The 3d dataset is further illustrated in the S1 Video.

The following acquisition and reconstruction parameters were used for the images in Fig 3: 46 partial fovs, partial fov matrix size of 96 by 128 by 59 (*x*,*y*,*z*) pixels further downsampled five-fold via averaging along the *z*-axis, 43.6 pixel overlap between partial fovs, *α* of 0.15, *β*_*i*_ of 0.04 ∀*i*, 10 iterations of the fista algorithm, 96 by 128 by 154 (*x*,*y*,*z*) final pixel matrix size, total imaging time of 10 min, and a fov of 4.5 cm by 3.5 cm by 7.5 cm (x,y,z).

In Fig 4, the proposed reconstruction is contrasted with the case of no dc recovery as well as the previous x-space algorithm using experimental mpi data from a coronary artery phantom. In the image with no dc recovery, the partial fov images were averaged together to form the image with no attempt to recover the lost dc information. There are obvious dropouts. When deconvolution is used, the background haze in the image is reduced; however, deconvolution has introduced one image signal dropout (marked with a dashed arrow).

The imaging parameters for Fig 4 were: 46 partial fovs, partial fov matrix size of 96 by 128 by 59 (*x*,*y*,*z*) pixels further downsampled six-fold via averaging along the *z*-axis, 43.6 pixel overlap between partial fovs, *α* of 0.05, *β*_*i*_ of 0.04 ∀*i*, 30 iterations of the fista algorithm, 96 by 128 by 129 (*x*,*y*,*z*) final pixel matrix size, total imaging time of 10 min, and a fov of 4.5 cm by 3.5 cm by 9.5 cm (x,y,z).

**Fig 6:**
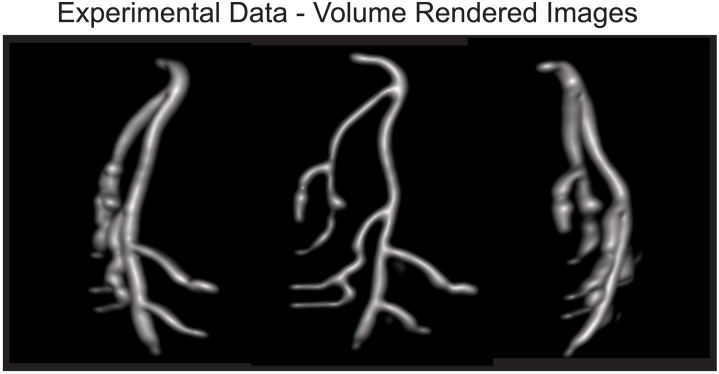
Experimental data of a coronary artery phantom from Fig 4 at different angles. The 3d volume-rendered datasets were reconstructed using the proposed method with deconvolution. The total imaging time was 10 min with a fov of 4.5 cm by 3.5 cm by 9.5 cm (*x*,*y*,*z*).

Fig 6 displays the data from the coronary artery phantom in Fig 4 with the proposed reconstruction at multiple angles of rotation to demonstrate the 3d nature of the dataset. The 3d dataset is further illustrated in the S2 Video. The images are volume rendered views with deconvolution.

**Fig 7:**
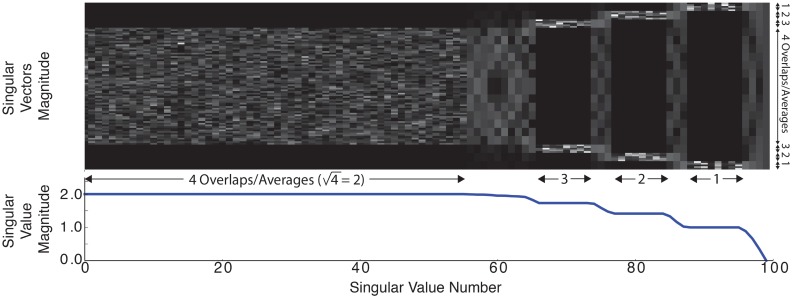
Singular values and right singular vectors, V, were calculated on A for a 1d problem where 15 pixels overlapped between adjacent partial fovs and the partial fov width was 20. The singular vectors represent the spatial *z*-axis and are shown in absolute value. The singular values demonstrate well-posed nature of the proposed reconstruction problem.

Fig 7 shows the singular values and right-singular vectors of the singular value decomposition (svd) calculated for the operator **A** to illustrate the conditioning of the proposed reconstruction. The operator was created for a 1d image reconstruction to allow the singular vectors to be shown easily. 15 pixels overlapped between adjacent partial fovs and the partial fov width was 20 pixels. As expected, there is a singular value of zero for the dc image component, which indicates that an image with only a dc component is in the nullspace of the operator. If the dc singular value is removed, the condition number of operator **A** is 6.

Table 1 details reduced memory requirements using matrix-free operators when reconstructing the coronary phantom images of Fig 4. All reconstruction was performed on a single core of a computer with four Xeon 5600 processors and 144GB ram. The conversion of **D** to a matrix-free operator reduced the reconstruction time 7-fold and reduced the storage requirement of the operator to negligible amounts (2 × 10^8^ fold reduction).

**Table 1:** Sparse matrix versus matrix-free operator computation time and ram requirements.

	Sparse Matrix	Matrix-Free Operator
ram	32 GB	0.000 000 2 GB
Computation Time	53 min	8 min

## Discussion

For clinical acceptance of any medical imaging system, developers must produce a robust system that gracefully handles noise and minimizes image artifacts [35, 36]. Here, we have designed an image reconstruction algorithm with these goals in mind.

In mpi, artifacts include banding and baseline drift. Banding artifacts manifest as ripples along the horizontal and vertical axes due to discontinuities between partial fovs. Haze occurs due to the long tails of the mpi psf and can be exacerbated by the reconstruction algorithm. Baseline drift also appears as a hazy background, but this is likely due to component heating in the mpi system.

The proposed reconstruction formulation improves resulting image robustness and remedies many of the artifacts seen in prior x-space algorithms. For example, Figs 3 and 4 show that the proposed reconstruction has improved conspicuity and reduced artifacts, including suppressing banding and minimizing haze. Because of the *a priori* information that the image is smooth, the banding artifacts do not occur in the images reconstructed via the optimization approach, which takes advantage of image smoothness along all image axes. The alpha term in the reconstruction optimization problem suppresses haze in the resulting images.

Reconstruction using the proposed formulation is well posed. The robustness of an optimization problem can be seen in the magnitude of the operator matrix’s singular values. To illustrate this, in Fig 7 we calculate the singular values and corresponding right singular vectors of a one-dimensional reconstruction using partial fov overlaps with similar properties as those used in the full 3d **A** matrix. We see that the singular value magnitude varies directly with the amount of signal averages in a reconstructed image region; the singular value plateaus are equal to the square root of the number of partial fov overlaps. For example, for singular value indices 1 to 64, each pixel in the central region is acquired four times in different partial fovs and these pixels have singular values of $\sqrt{4}=2$ . Note the region of variation (marked with 4 averages along the *y*-axis) in the singular vectors image corresponds to the section of four overlapping partial fovs where the singular value magnitude is 2.

The proposed algorithm can recover the dc information within a partial fov, but there is no *a priori* information to recover the overall dc value of the image. This problem is common to all mpi techniques that filter the signal direct-feedthrough. Note in Fig 7 that the right-most singular value of the svd is zero; the dc value is in the null space of **A**. The minimum dc value is selected out of the null space by the optimization problem regularization term, which will be correctly selected if there is at least a single pixel value of mnp concentration within each line in the fov. Images taken with mpi are sparse and anatomical structures are tortuous by nature, meaning images contain many zero values. Correct selection can be guaranteed by ensuring there is no tracer at one edge of the fov during scan prescription. Furthermore, even with this condition not guaranteed, tests have indicated that the proposed algorithm still performs well.

A reconstruction algorithm should not cause noise gain. As seen in Fig 7, the 1d svd contains a small number of singular values less than 1. These singular values represent a noise gain but the smoothness and Tikhonov terms suppress their noise amplification contributions. Furthermore, the very low frequency and straight line input distributions that would map to these singular values are not typically found in biological samples.

Beyond reconstruction, svd analysis can also be applied to the design of mpi pulse sequences. Inspection of Fig 7 indicates that greater snr efficiency may be achieved by adding additional acquisitions near the edge of the fov to better condition the reconstruction. A larger drive field will create more image overlap and thus more averaging but will not necessarily greatly improve the conditioning of the reconstruction. The same can be said about using a finer shift field pattern.

**Fig 8:**
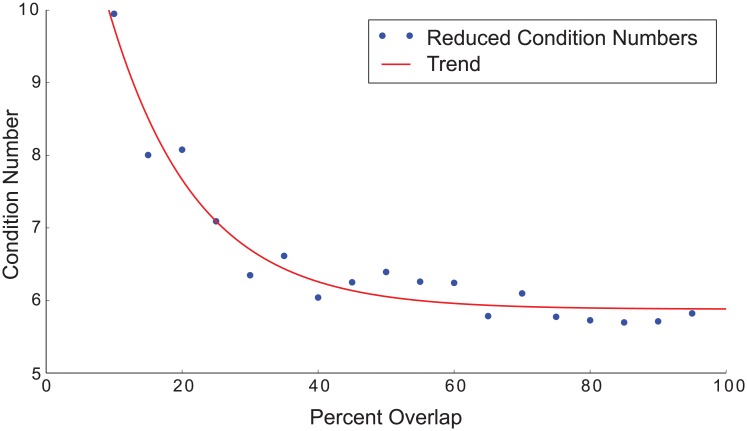
Condition number variation with overlap. The condition number is calculated on the matrix **A** with the dc singular vector removed (reduced **A**) for a 1d problem with a partial fov width of 20. The trend curve is a least-squares fit to the calculated condition numbers and illustrates the general trend of improved condition number with increased partial fov overlap.

Reducing the overlap in the pulse sequence does not significantly increase the condition number until the overlap becomes small (see Fig 8). This indicates that reducing the overlap does not pose significant reconstruction problems until the overlap is only a small portion of the partial fov. Though the conditioning does not significantly decrease, reduced averaging due to reduced overlap will increase the noise seen in images as discussed above.

The above svd analysis demonstrates that image reconstruction via the proposed optimization method is robust. Furthermore, the proposed method has been shown to produce fewer artifacts than the the previous x-space approach. We anticipate that improved mpi reconstruction techniques such as optimized 3d reconstruction will be crucial for the long term acceptance of mpi in the clinic. In addition, we believe that these methods, along with advances in hardware and mnp design, will be important for improved image quality in the future.

The proposed reconstruction technique contrasts with deconvolution, which if not used carefully and judiciously can degrade snr and introduce artifacts such as signal dropouts. This effect is seen in Fig 4, where there is one dropout in the deconvolved image that is not present in the actual reconstructed image (marked with an arrow). However, deconvolution is able to reduce the haze present in the reconstructed image when applied minimally. It is thus vital that the benefits of deconvolution, such as reduction of haze, be balanced with the potential for introducing artifacts such as signal dropouts and ringing.

The proposed reconstruction technique is fast and scales well. With matrix-free techniques, reconstruction occurs in eight minutes for the full 3d volume using only a single processor. Moreover, many techniques could speed the solution of the optimization problem. Parallel processing techniques on multiple core cpus or gpus could be used. Also, for real time imaging, a prior reconstructed frame can be used to seed the optimization problem for rapid convergence.

The proposed optimization approach is extensible in many ways. In general, new *a priori* information can be incorporated into the reconstruction formulation. The proposed reconstruction can be modified for other mpi trajectories, to add multiple simultaneous drive and receive channels, and to include filtered backprojection for ffl mpi systems. Expansion of the formulation to include filtering and gridding steps of x-space mpi can be explored. Relaxation affects could be added to the formulation to improve reconstruction and enable new applications. Compressed sensing approaches can be explored by reformulating the optimization problem and including objective terms such as sparsity transforms: wavelet transforms, discrete cosine transforms, or Chebyshev transforms. Many of these techniques have been used in mri and ct to improve image quality.

## Conclusion

We reformulated dc recovery in x-space reconstruction as a 3d optimization problem. This represents the first implementation of x-space reconstruction to take advantage of information along axes perpendicular to the excitation axis during dc recovery on an ffp mpi system. The reconstruction uses robust a *a priori* information, non-negativity and image smoothness, to improve image quality. We applied the reconstruction algorithm to measured data and demonstrated improved robustness (less banding and haze artifacts) compared to our previous work. The framework developed here has improved flexibility over our prior 1d-at-a-time technique, and shows promise for future work in mpi, including generalized trajectories in x-space, projection reconstruction, filtering incorporation, and compressed sensing.

## Supporting Information

S1 Video Experimental data of a double helix phantom. A video exported from OsiriX (Pixmeo SARL, Bernex, Switzerland) illustrates the 3d dataset of Fig 3 in rotated maximum intensity projection.
(MP4)

S2 Video Experimental data of a coronary artery phantom. A video exported from OsiriX (Pixmeo SARL, Bernex, Switzerland) illustrates the 3d dataset of Figs 4 and 6 in rotated maximum intensity projection.
(MP4)
